# Harnessing Facebook to Investigate Real-World Mentions of Adverse Events of Glucagon-Like Peptide-1 Receptor Agonist (GLP-1 RA) Medications: Observational Study of Facebook Posts From 2022 to 2024

**DOI:** 10.2196/73619

**Published:** 2025-07-24

**Authors:** Amrutha S Alibilli, Vidur Jain, Heran Mane, Xiaohe Yue, Alexandria Ratzki-Leewing, Junaid S Merchant, Shaniece Criss, Quynh C Nguyen, Rozalina G McCoy, Thu T Nguyen

**Affiliations:** 1 Department of Epidemiology and Biostatistics, University of Maryland, College Park, 4254 Stadium Drive, College Park, 20742, United States, 1 301-405-6589; 2 Division of Gerontology, Department of Epidemiology and Public Health, University of Maryland School of Medicine, Baltimore, MD, United States; 3 University of Maryland Institute for Health Computing, North Bethesda, MD, United States; 4 Department of Health Sciences, Furman University, Greenville, SC, United States; 5 National Institute of Nursing Research, National Institutes of Health, Bethesda, MD, United States; 6 Division of Endocrinology, Diabetes, and Nutrition, Department of Medicine, University of Maryland School of Medicine, Baltimore, MD, United States

**Keywords:** social media, semaglutide, tirzepatide, Ozempic, Wegovy, Mounjaro, Zepbound, GLP-1 RAs, adverse events, obesity management, weight loss medication

## Abstract

**Background:**

In recent years, there has been a dramatic increase in the popularity and use of glucagon-like peptide-1 receptor agonists (GLP-1 RAs) for weight loss. As such, it is essential to understand users’ real-world discussions of short-term, long-term, and co-occurrent adverse events associated with currently used GLP-1 RA medications.

**Objective:**

This study aims to quantitatively analyze temporal and co-occurrent GLP-1 RA adverse event trends through discussions of GLP-1 RA weight loss medications on Facebook from 2022 to 2024.

**Methods:**

We collected 64,202 Facebook posts (59,293 posts after removing duplicate posts) from January 1, 2022, to May 31, 2024, through CrowdTangle, a public insights tool from Meta. Using English language social media posts from the United States, we examined discussions of adverse event mentions for posts referencing 7 GLP-1 RA weight loss product categories (ie, semaglutide, Ozempic, Wegovy, tirzepatide, Mounjaro, Zepbound, and GLP-1 RA as a class). All analyses were conducted using Python (version 3; Python Software Foundation) in a Google Colab environment.

**Results:**

Temporal time series analysis revealed that the GLP-1 RAs’ adverse event mentions on social media aligned with several key events: the Food and Drug Administration’s approval of Wegovy for pediatric weight management in December 2022, increased media coverage in August 2023, celebrity endorsement in December 2023, and Medicare Part D coverage expansion for weight loss medications in March 2024. Gastrointestinal (GI)–related adverse events (general term) were most prevalent for posts mentioning the GLP-1 RA class (210/4885, 4.30%) and Mounjaro (241/4031, 5.98%). In contrast, the most prevalent adverse event mentions noted for tirzepatide were headache (78/4202, 1.86%) and joint pain (71/4202, 1.69%). Hypertension (13/1769, 0.73%) was frequently mentioned in Zepbound posts, while pancreatitis was commonly associated with Mounjaro posts (44/4031, 1.08%), and 2.85% (139/4885) of posts broadly referring to the GLP-1 RA class. Furthermore, an integrated node network analysis revealed 3 distinct GLP-1 RA adverse events–mentioned clusters: cluster 1 (purple) contained allergies, anxiety, depression, chronic obstructive pulmonary disease, fatigue, fever, hypertension, indigestion, insomnia, gastroesophageal reflux disease, hives, swelling, restlessness, and seizures. Cluster 2 (pink) contained constipation, dehydration, headache, diarrhea, dizziness, hypoglycemia, sweating, and jaundice. Cluster 3 (brown) contained GI symptoms, such as nausea, pancreatitis, rash, and vomiting. The GI symptoms, such as nausea, vomiting, pancreatitis, diarrhea, and indigestion, were strongly associated together (≥100 co-occurrence mentions), while the mentioned neurological symptoms, such as anxiety, depression, and insomnia, were highly correlated with each other (50‐100 co-occurrence mentions).

**Conclusions:**

This social media study highlights the adverse event mention patterns for posts referencing GLP-1 RA medications. While further research is needed to rigorously examine and validate these findings, this study demonstrates the importance of monitoring social media discussions to predict novel, underreported, or rare drug adverse events, thereby improving patient care, clinical research, and health policy interventions.

## Introduction

In recent years, there has been a dramatic increase in the popularity and use of glucagon-like peptide-1 receptor agonists (GLP-1 RAs) for the treatment of type 2 diabetes and obesity [1]. The Food and Drug Administration (FDA) first approved Ozempic (semaglutide) and Mounjaro (tirzepatide) for the management of type 2 diabetes in December 2017 and May 2022, respectively. Wegovy (semaglutide) and Zepbound (tirzepatide) were then approved for the management of obesity and overweight in June 2021 and November 2023, respectively. Given that nearly 38 million US adults are living with diabetes and more than 100 million have obesity, the increased demand for these drugs has contributed to an alarming global shortage of medications for people in need [2,3]. Furthermore, the low availability, high costs, and inconsistent and inadequate insurance coverage of GLP-1 RAs have contributed to the rise of compounded versions of GLP-1 RAs. However, while often lower in cost and more readily available (including without a prescription), compounded GLP-1 RAs have unknown dosage, safety, and efficacy profiles [4,5].

An important consideration for GLP-1 RAs’ use is their adverse event profile, including significant GI discomfort and other potential physical and psychological symptoms [6]. GLP-1 RAs’ functions include stimulating glucose-dependent insulin secretion, inhibiting glucagon secretion, and delaying gastric emptying [7]. These mechanisms of action contribute to reducing blood glucose levels and facilitating weight loss. However, by reducing patients’ appetite and nutritional intake, GLP-1 RAs may lead to malnutrition, dehydration, and rapid loss of both fat and lean (ie, muscle and skeletal) body mass [8]. Previous clinical studies have discovered prevalent adverse events associated with GLP-1 RAs, including GI symptoms, such as nausea, diarrhea, vomiting, and reduced appetite [9]. Others revealed notable mental health adverse events, including insomnia, anxiety, and depression [6,10]. Importantly, the individual experience with medications—concerning effectiveness, tolerability, and safety—may differ among those taking medications in the real world compared with those enrolled and studied in clinical trials, and some events may be too infrequent to be detected in clinical trials. Moreover, rare adverse events may not be ascertained in clinical trial protocols. Therefore, further research is necessary to understand the complex adverse events of GLP-1 RAs on physical and mental health conditions experienced and reported by individuals consuming these medications in contemporary practice.

Since the most potent GLP-1 RAs (including the dual glucose-dependent insulinotropic polypeptide and GLP-1 RA, tirzepatide) were introduced into practice recently, it is important to monitor for emerging adverse events of these medications [11]. Clinical studies identify many of GLP-1 RAs’ adverse events, but they cannot capture the totality of adverse events experienced by and important to patients. For instance, clinical studies’ sample size and duration of follow-up are often limited, reducing the likelihood of detecting rare or long-term adverse events. Individuals taking GLP-1 RAs also differ from those included in clinical trials as they likely have different clinical profiles, lifestyles, environmental contexts, and dietary and physical activity cointerventions. Furthermore, the robustness of education and clinical monitoring available before and while taking GLP-1 RA medications contributes to individuals’ perceptions of the medications. It is also essential to monitor adverse events of compounded GLP-1 RA products, whose safety profiles are expected to differ from the FDA-approved products and are largely unknown at this time.

Social media platforms provide a relatively untapped, extensive source of public discussion from which health professionals can develop targeted interventions. A meta-analysis study analyzed social media pharmacovigilance studies and FDA reports and found that more adverse event discussions on social media corresponded to a higher hazard and faster drug product recall [12]. Therefore, social media may reveal invaluable insights regarding mentioned adverse events that may not be presented in other clinical or observational studies.

Building upon prior findings, this social media–based study aims to use advanced data extraction techniques and conduct quantitative analyses to investigate mentioned GLP-1 RA adverse event phenotypes. Our approach harnesses social media data as a complement to traditional clinical and pharmacovigilance data sources to identify user-reported mentions of adverse events for posts referencing GLP-1 RAs. Additionally, we examine temporal trends of social media GLP-1 RA discussions to identify factors that drive their popularity from Oprah Winfrey’s endorsement of GLP-1 RAs [13] to Medicare’s coverage expansion [14]. Finally, we identify clusters of co-mentioned adverse events, introducing a novel way to characterize adverse event interconnections and risk profiles. Quantitative temporal analyses are vital to highlight possible unreported GLP-1 RA adverse events, providing timely information to inform community-driven interventions to promote public health awareness about GLP-1 RA use.

## Methods

### Data Source

We collected Facebook posts through CrowdTangle, a public insights tool from Meta [15] and engagement metrics (eg, views and post likes). CrowdTangle has been discontinued. Currently available tools for researchers include Meta Content Library and Content Library API [16]. CrowdTangle provides data from public accounts, including 7 million Facebook pages, groups, and verified profiles. Data collection can be refined by specifying various parameters such as time frame, specific pages, groups or profiles, keywords or tags, language, geolocation, and presence of memes. The returned data include posts, image URLs, image caption text, date of the post, and engagement metrics (eg, views and post likes).

### Data Preparation (Sample and Inclusion Criteria)

Using CrowdTangle, we obtained and processed Facebook English language posts by US-based users between January 1, 2022, and May 31, 2024, that mentioned weight loss medications, specifically “Semaglutide/semaglutide,” “Ozempic/Ozempic/ozempy,” “Wegovy/wegovy,” “Tirzepatide/tirzepatide,” “Mounjaro/mounjaro,” “Zepbound/zepbound,” and “GLP-1/GLP-1s/GLP-1 agonist/GLP-1 RAs.” To limit our collection to US-based discussions on weight loss medications, we leveraged CrowdTangle’s “Page Admin Country” filtering parameter. This field identifies the country of the primary administrators of Facebook Pages. Because the filter was not available for Facebook Groups, we collected posts derived solely from Facebook Pages where the United States was listed as the “Page Admin Country. Among these posts, we identified those that mentioned any potential short-term or long-term adverse events, defined as any undesirable experience associated with a drug or treatment [17] (Table 1). The list of adverse events was determined based on past literature, clinical expertise, and the SIDER database version 4.1, which cataloged 5868 documented adverse events for 1430 commercially available drugs [18]. To examine the magnitude of adverse events mentioned for weight loss medications, duplicate posts with the same message were filtered out.

**Table 1.** List of adverse events associated with different weight loss medications.

Adverse event category	Adverse event
Gastrointestinal	Gastrointestinal (general term) Nausea Vomiting Diarrhea Constipation Abdominal pain Loss of appetite Indigestion Jaundice Pancreatitis Gallbladder issues Gastroesophageal reflux disease Liver damage
Neuromuscular	Headache Dizziness Seizures Muscle cramps Tingling sensation Joint pain Muscle weakness Back pain
Cardiovascular and respiratory	Persistent cough Swelling Increased heart rate Shortness of breath Hypertension Chronic obstructive pulmonary disease Blood clots Heart palpitations
Dermatologic and immunologic	Rash Sweating Hives Sore throat Fever Dry mouth Hair loss Allergies
Endocrine and metabolic	Elevated blood sugar levels Irregular menstrual cycle Thyroid tumor Erectile dysfunction Kidney damage Hypoglycemia
Mental health and behavioral	Depression Anxiety Mood swings Insomnia Restlessness
General symptoms	Vision changes Fatigue Dehydration

### Statistical Analysis

Descriptive analyses were conducted using Python (version 3) in a Google Colab environment. For each weight loss medication, we calculated the frequency and percentage of adverse events mentioned—calculated as the total mention of a certain adverse events over the total number of posts for a particular weight loss medication. To examine temporal trends, we recorded the total frequency of adverse events by calendar month. We plotted frequency and percentage of adverse events mentions from January 2022 to May 2024 to assess for changes in the mentions of adverse events for four specific events: (1) FDA approval of Wegovy for weight management in children aged 12‐17 years in December 2022 [19], (2) increased media coverage of GLP-1 RA medications on TikTok and other platforms in August of 2023 [20], (3) Oprah Winfrey’s endorsement of weight loss medications in December 2023 [14], and (4) expanded FDA indication for Wegovy for the prevention of cardiovascular events and mortality in adults with obesity and cardiovascular disease in March 2024, paving the way for Medicare coverage of weight management medications for reduction of cardiovascular events [21].

To investigate the co-occurrence of adverse events mentioned, a cluster analysis was conducted. Clusters were formed based on the Louvain community detection algorithm using the Python library (python-louvain) [22], which can detect communities (via modularity optimization) and subsequently improve the quality of community structures [23]. A spring layout algorithm for positioning with k=7.8 and the number of iterations=100 was applied to arrange the nodes to effectively repel them to minimize overlap and enhance the clarity of the node-network structure [24]. Using this method, we constructed a network of adverse events mentioned where 2 nodes mentioned in the same post are connected by an edge. A cluster was based on the co-occurrence of mentioned adverse events. The size of each node reflects the frequency of the mentions, while the width of each edge (thickness of the lines) corresponds to its weight, indicating the strength of co-occurrence between comentioned adverse events.

### Ethical Considerations

This study was determined to be not human participant research by the University of Maryland College Park Institutional Review Board (2072551-1). In addition, the social media posts were anonymized, upholding user privacy.

## Results

### Descriptive Patterns of Adverse Events

Table S1 in Multimedia Appendix 1 shows the FDA-reported adverse events of the drugs examined in this study with their brand name, generic name, and approved indication for the drug. We collected 64,202 (59,293 posts after removing duplicates) posts that included a discussion of the 7 weight loss products. Of these posts, 28.9% (17,146/59,293) on semaglutide, 31.6% (18,733/59,293) on Ozempic, 14.4% (8527/59,293) on Wegovy, 7.1% (4202/59,293) on tirzepatide, 6.8% (4031/59,293) on Mounjaro, 3.0% (1769/59,293) on Zepbound, and 8.5% (4885/59,293) referenced the GLP-1 RA class without mentioning a specific drug. Among all the posts, 13.8% (8171/59,293) mentioned adverse events (Table 2). Table 2 shows the counts and percentages of all posts about each medication that mentions specific adverse events. For example, nausea is mentioned in 3.23% (185/4885) of posts referencing GLP-1. Within each column of the different medications, adverse event mentions are depicted in decreasing frequency within broad categories of adverse events such as “Gastrointestinal” or “Mental Health and Behavior,” where the highest percentage within the category is near the top, and the lowest percentage is near the bottom. The percentages in boldface represent the highest frequency within each column.

**Table 2.** Frequency and percentage of adverse event mentions by weight loss medications^a^.

	Adverse events	GLP-1^b^	Semaglutide	Ozempic	Wegovy	Mounjaro	Tirzepatide	Zepbound
	Total posts	4885	17,146	18,733	8527	4031	4202	1769
Category	Total number of adverse event mentions within drug posts	1138	1486	2579	1495	868	436	169
Gastrointestinal	Gastrointestinal (general term), n (%)	**210 (4.30)^c^**	95 (0.55)	**488 (2.61)**	**296 (3.47)**	**241 (5.98)**	19 (0.45)	16 (0.90)
	Nausea, n (%)	158 (3.23)	**197 (1.15)**	317 (1.69)	175 (2.05)	86 (2.13)	48 (1.14)	**23 (1.30)**
	Vomiting, n (%)	113 (2.31)	99 (0.58)	308 (1.64)	228 (2.67)	153 (3.80)	17 (0.40)	11 (0.62)
	Pancreatitis, n (%)	139 (2.85)	52 (0.30)	165 (0.88)	91 (1.07)	44 (1.09)	12 (0.29)	7 (0.40)
	Constipation, n (%)	59 (1.21)	64 (0.37)	143 (0.76)	82 (0.96%)	38 (0.94)	26 (0.62)	16 (0.90)
	Diarrhea, n (%)	64 (1.31)	59 (0.34)	155 (0.83)	83 (0.97)	36 (0.89)	19 (0.45)	12 (0.68)
	Abdominal pain, n (%)	51 (1.04)	22 (0.13)	112 (0.60)	34 (0.40)	19 (0.47)	6 (0.14)	2 (0.11)
	Gallbladder issues, n (%)	13 (0.27)	4 (0.02)	61 (0.33)	36 (0.42)	3 (0.07)	1 (0.02)	1 (0.06)
	Indigestion, n (%)	2 (0.04)	5 (0.03)	6 (0.03)	0 (0.00)	5 (0.12)	0 (0.00)	0 (0.00)
	Loss of appetite, n (%)	4 (0.08)	2 (0.01)	5 (0.03)	4 (0.05)	0 (0.00)	0 (0.00)	0 (0.00)
	GERD^d^, n (%)	5 (0.10)	2 (0.01)	4 (0.02)	1 (0.01)	1 (0.02)	0 (0.00)	0 (0.00)
	Liver damage, n (%)	0 (0.00)	1 (0.01)	1 (0.01)	0 (0.00)	0 (0.00)	0 (0.00)	0 (0.00)
	Jaundice, n (%)	0 (0.00)	1 (0.01)	0 (0.00)	0 (0.00)	0 (0.00)	0 (0.00)	0 (0.00)
Mental health and behavior	Depression, n (%)	42 (0.86)	151 (0.88)	165 (0.88)	134 (1.57)	19 (0.47)	13 (0.40)	9 (0.62)
	Anxiety, n (%)	39 (0.80)	59 (0.34)	70 (0.37)	36 (0.42)	19 (0.47)	13 (0.31)	9 (0.51)
	Mood swings, n (%)	3 (0.06)	24 (0.14)	15 (0.08)	14 (0.16)	2 (0.05)	3 (0.07)	0 (0.00)
	Insomnia, n (%)	4 (0.08)	7 (0.04)	6 (0.03)	1 (0.01)	5 (0.12)	2 (0.05)	0 (0.00)
	Restlessness, n (%)	1 (0.02)	0 (0.00)	0 (0.00)	0 (0.00)	0 (0.00)	0 (0.00)	0 (0.00)
Dermatologic and immunologic	Hair loss, n (%)	48 (0.98)	50 (0.29)	153 (0.82)	77 (0.90)	80 (1.98)	15 (0.36)	17 (0.96)
	Rash, n (%)	8 (0.16)	51 (0.30)	25 (0.13)	5 (0.06)	6 (0.15)	4 (0.10)	1 (0.06)
	Fever, n (%)	2 (0.04)	5 (0.03)	7 (0.04)	5 (0.06)	3 (0.07)	1 (0.02)	2 (0.11)
	Sweating, n (%)	3 (0.06	9 (0.05)	13 (0.07)	4 (0.05)	2 (0.05)	1 (0.02)	0 (0.00)
	Allergies, n (%)	1 (0.02)	14 (0.08)	7 (0.04)	3 (0.04)	2 (0.05)	1 (0.02)	0 (0.00)
	Hives, n (%)	6 (0.12)	1 (0.01)	7 (0.04)	1 (0.01)	1 (0.02)	1 (0.02)	0 (0.00)
	Sore throat, n (%)	2 (0.04)	0 (0.00)	3 (0.02)	3 (0.04)	0 (0.00)	0 (0.00)	0 (0.00)
	Dry mouth, n (%)	0 (0.00)	1 (0.01)	0 (0.00)	0 (0.00)	0 (0.00)	1 (0.02)	0 (0.00)
Neuromuscular	Headache, n (%)	21 (0.43)	111 (0.65)	22 (0.12)	13 (0.15)	**7 (0.17)**	**78 (1.86)**	3 (0.17)
	Joint pain, n (%)	10 (0.20)	97 (0.57)	4 (0.02)	2 (0.02)	4 (0.10)	71 (1.69)	1 (0.06)
	Dizziness, n (%)	8 (0.16)	15 (0.09)	19 (0.10)	16 (0.19)	2 (0.05)	0 (0.00)	1 (0.06)
	Muscle weakness, n (%)	2 (0.04)	2 (0.01)	2 (0.01)	2 (0.02)	2 (0.05)	1 (0.02)	0 (0.00)
	Back pain, n (%)	0 (0.00)	7 (0.04)	1 (0.01)	0 (0.00)	2 (0.05)	0 (0.00)	0 (0.00)
	Seizures, n (%)	0 (0.00)	4 (0.02)	6 (0.03)	0 (0.00)	0 (0.00)	0 (0.00)	0 (0.00)
	Muscle cramps, n (%)	0 (0.00)	3 (0.02)	0 (0.00)	0 (0.00)	0 (0.00)	0 (0.00)	0 (0.00)
	Tingling sensation, n (%)	0 (0.00)	1 (0.01)	0 (0.00)	0 (0.00)	0 (0.00)	0 (0.00)	0 (0.00)
General symptom	Fatigue, n (%)	29 (0.59)	111 (0.65)	65 (0.35)	47 (0.55)	21 (0.52)	33 (0.79)	18 (1.02)
	Vision changes, n (%)	4 (0.08)	3 (0.02)	4 (0.02)	3 (0.04)	3 (0.07)	2 (0.05)	2 (0.11)
	Dehydration, n (%)	4 (0.08)	12 (0.07)	8 (0.04)	3 (0.04)	2 (0.05)	0 (0.00)	0 (0.00)
Cardiovascular and respiratory	Hypertension, n (%)	32 (0.66)	53 (0.31)	24 (0.13)	25 (0.29)	11 (0.27)	21 (0.50)	13 (0.73)
	Persistent cough, n (%)	0 (0.00)	0 (0.00)	22 (0.12)	0 (0.00)	22 (0.55)	0 (0.00)	0 (0.00)
	Swelling, n (%)	1 (0.02)	24 (0.14)	10 (0.05)	7 (0.08)	2 (0.05)	4 (0.10)	0 (0.00)
	COPD^e^, n (%)	4 (0.08)	0 (0.00)	7 (0.04)	0 (0.00)	1 (0.02)	0 (0.00)	0 (0.00)
	Blood clots, n (%)	4 (0.08)	2 (0.01)	2 (0.01)	1 (0.01)	1 (0.02)	1 (0.02)	0 (0.00)
	Increased heart rate, n (%)	0 (0.00)	2 (0.01)	2 (0.01)	3 (0.04)	1 (0.02)	1 (0.02)	0 (0.00)
	Heart palpitations, n (%)	0 (0.00)	3 (0.02)	2 (0.01)	0 (0.00)	2 (0.05)	1 (0.02)	0 (0.00)
	Shortness of breath, n (%)	0 (0.00)	1 (0.01)	0 (0.00)	4 (0.05)	0 (0.00)	0 (0.00)	0 (0.00)
Endocrine and metabolic	Hypoglycemia, n (%)	29 (0.59)	31 (0.18)	80 (0.43)	33 (0.39)	9 (0.22)	7 (0.17)	2 (0.11)
	Thyroid tumor, n (%)	8 (0.16)	15 (0.09)	54 (0.29)	20 (0.23)	9 (0.22)	3 (0.07)	1 (0.06)
	Erectile dysfunction, n (%)	1 (0.02)	10 (0.06)	2 (0.01)	2 (0.02)	0 (0.00)	5 (0.12)	0 (0.00)
	Kidney damage, n (%)	4 (0.08)	4 (0.02)	5 (0.03)	1 (0.01)	2 (0.05)	1 (0.02)	0 (0.00)
	Elevated blood sugar levels, n (%)	0 (0.00)	0 (0.00)	1 (0.01)	0 (0.00)	0 (0.00)	0 (0.00)	0 (0.00)
	Irregular menstrual cycles, n (%)	0 (0.00)	0 (0.00)	1 (0.01)	0 (0.00)	0 (0.00)	0 (0.00)	0 (0.00)

a Percentage = (Number of posts mentioning the adverse event for a medication/Total number of posts about the medication) × 100. The values in boldface indicate the most commonly mentioned adverse event for the particular GLP-1 RA category.

b GLP-1: glucagon-like peptide-1.

c The values in boldface indicate the most commonly mentioned adverse event for the particular GLP-1 RA category.

d GERD: gastroesophageal reflux disease.

e COPD: chronic obstructive pulmonary disease.

Distinct GLP-RA adverse events mention patterns emerged. General (nonspecified) GI concerns were among the most frequently reported, representing 4.3% (210/4885) of general GLP-1 RA posts and 6.0% (241/4031) and 3.5% (296/8527) of posts referencing Mounjaro and Wegovy, respectively (Table 2). A lower percentage of GI issues were reported in posts mentioning Ozempic (488/18,733, 2.6%), semaglutide (95/17,146, 0.5%), tirzepatide (19/4202, 0.5%), and Zepbound (16/1769, 0.9%). Nausea was among the most commonly reported specific symptoms, appearing in 3.2% (158/4885) of posts about general GLP-1 RA use, 2.1% (86/4031) of posts about Mounjaro and Wegovy each, 1.7% (317/18,733) about Ozempic, and 1.3% (23/1769) of posts about Zepbound; it was mentioned in approximately 1% (197/17,146) of semaglutide and tirzepatide posts. Vomiting was also mentioned in 3.8% (153/4031) of Mounjaro posts, 2.7% (228/8527) of Wegovy posts, 2.3% (113/4885) of general GLP-1 RA posts, 1.6% of Ozempic posts (308/18,733), and 0.6% of semaglutide (99/17,146) and Zepbound (11/1769) posts each. Pancreatitis was a concern that appeared in 2.9% (139/4885) of general GLP-1 RAs posts.

Several other adverse events were referenced in posts. Depression was noted across various GLP-1 RAs, although it was less prevalent than general GI concerns or nausea and vomiting (Table 2). Wegovy posts mentioned depression 1.6% (134/8527) of the time, while general GLP-1 RAs (42/4885), Ozempic (165/18,733), and semaglutide (151/17,146) posts discussed depression 0.9% of the time. Depression was less often noted in Zepbound (9/1769, 0.6%), Mounjaro (19/4031, 0.5%), and tirzepatide (13/4202, 0.4%)-related posts. Hair loss was more prominent in Mounjaro posts (80/4031, 2.0%) than in general GLP-1 RA (48/4885) or Zepbound (17/1769) posts, which were discussed in 1.0% of posts each. Headaches (78/4202) and joint pain (71/4202) appeared in 2.0% of tirzepatide posts, compared with 0.6% (111/17,146) and 0.7% (97/17,146) of semaglutide posts. Fatigue was also reported across all weight loss medications. Table S2 in Multimedia Appendix 1 shows the percentage of posts that mention each adverse event by medication. For example, 1.69% (317/18,733) of posts mentioning Ozempic adverse events include “nausea.” Figure S1 in Multimedia Appendix 1 displays the prominent adverse events frequencies (total≥75) for the weight loss medications.

### Temporal Trends With Key Events

**Figure 1.**
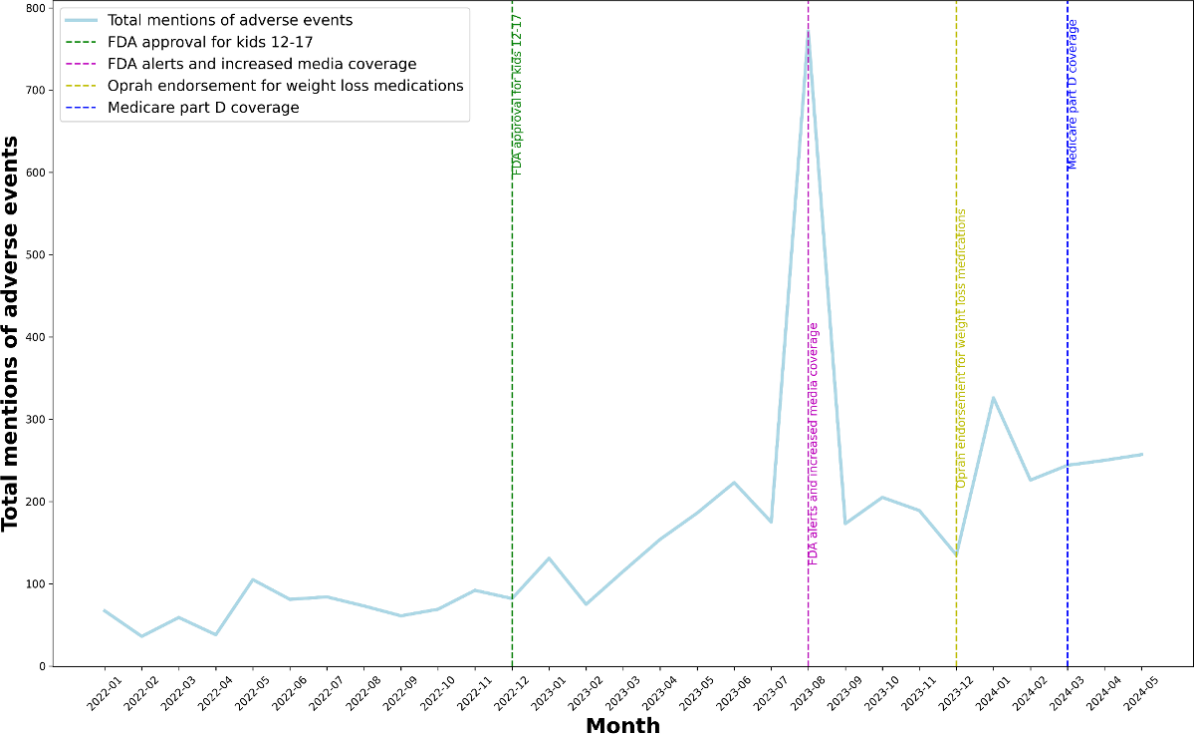
Temporal time series analysis of mention of all adverse events from January 2022 to May 2024. FDA: Food and Drug Administration.

We examined the potential impact of several key events occurring during the temporal analysis period on observed side effect mention trends (Figure 1). In December 2022, the FDA approved Wegovy for the treatment of obesity in children aged 12‐17 years [19]. This event was followed by increased media coverage in August 2023 that highlighted potential adverse event mentions associated with these drugs and interactions with other medications, coinciding with a surge in media coverage across social media platforms including Reddit, TikTok, Instagram, Facebook, and Twitter [19]. At the end of 2023, Oprah Winfrey publicly endorsed the use of weight loss medications (December 14, 2023) [14], while Medicare Part D expanded its coverage for weight loss medications starting in March 2024 [21]. Figure S2 and Table S3 in Multimedia Appendix 1 show the percentage of adverse events mentioned to total drug mentions over time. Of note, although the number of adverse events mentioned has increased over time, the percentage of posts that mention adverse events has decreased over time (Figure S2 in Multimedia Appendix 1), indicating changes in the composition of these discussions from adverse events to other topics related to these medications.

### Co-Occurrence Network Trends

**Figure 2.**
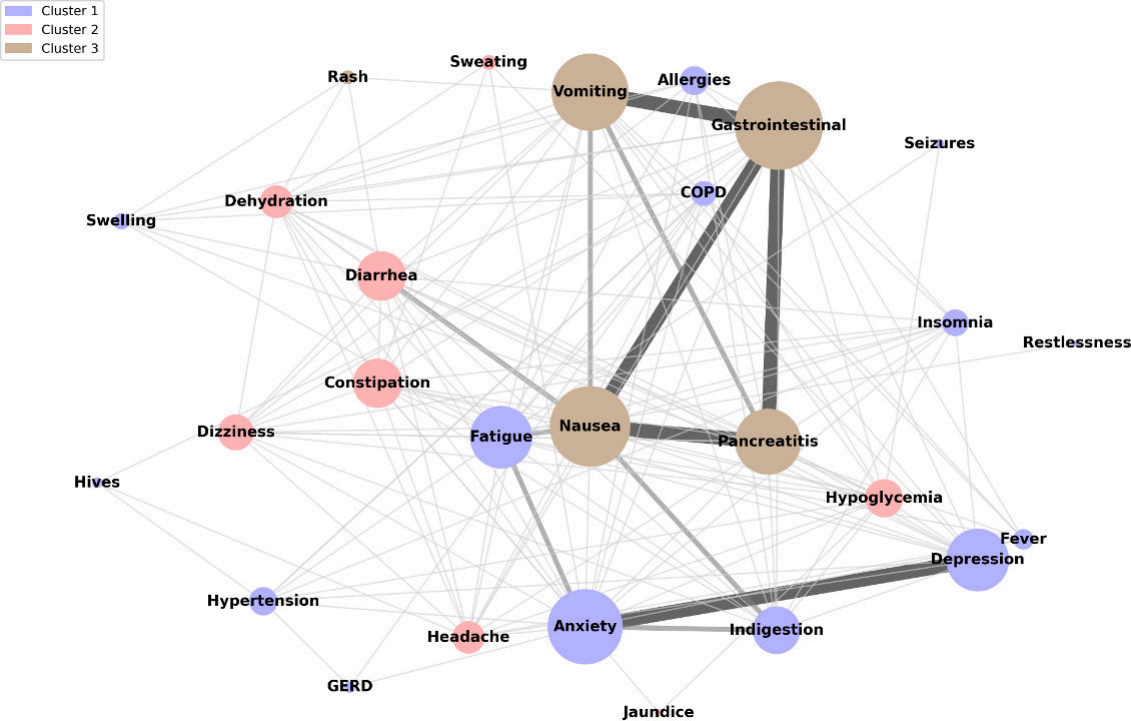
Network analysis of adverse events co-occurrences and community clusters. COPD: chronic obstructive pulmonary disease; GERD: gastroesophageal reflux disease.

In a network analysis of noted adverse events for posts referencing these medications, a total of 2618 adverse event co-occurrence interactions and 27 nodes (circles representing adverse events) were identified from the dataset. As shown in Figure 2, 3 distinct communities were formed, each representing a grouping of adverse events mentioned with similar characteristics based on their high-frequency co-occurrence patterns. Cluster 1 (purple) contains allergies, anxiety, depression, chronic obstructive pulmonary disease, fatigue, fever, hypertension, indigestion, insomnia, gastroesophageal reflux disease, hives, swelling, restlessness, and seizures. Cluster 2 (pink) contains constipation, dehydration, headache, diarrhea, dizziness, hypoglycemia, sweating, and jaundice. Cluster 3 (brown) contains GI symptoms, such as nausea, pancreatitis, rash, and vomiting. Interactions with a co-occurrence count of ≥100 were visualized with thick lines, while those with counts between 50 and 100 were shown with a moderately thick line, and interactions with counts <50 were represented by the thinnest lines. As depicted in Figure 2, nausea, vomiting, pancreatitis, and GI distress are connected by thick lines, reflecting their strong co-occurrence. Anxiety and depression are also highly correlated. Next, the intermediately thick lines reveal moderately strong associations between fatigue, anxiety, indigestion, nausea, diarrhea, vomiting, and pancreatitis. The most common co-occurrences are GI and vomiting (298), GI and pancreatitis (152), GI and nausea (132), anxiety and depression (210), and nausea and pancreatitis (124). Moderate co-occurrences include anxiety and indigestion (96), anxiety and fatigue (76), and fatigue and nausea (74).

## Discussion

### Principal Findings

This study used Facebook data to examine the adverse events discussed on the social media platform with respect to different GLP-1 RAs commonly used for weight management from January 2022 to June 2024. Semaglutide and Ozempic (the brand of semaglutide FDA-approved for the treatment of type 2 diabetes) were the most mentioned products, while Wegovy (the brand of semaglutide FDA-approved for the treatment of obesity), tirzepatide, Mounjaro (the brand of tirzepatide FDA-approved for the treatment of type 2 diabetes), Zepbound (the brand of tirzepatide FDA-approved for the treatment of obesity), and the overall GLP-1 RA class were discussed less often, reflecting the public’s attention to Ozempic as a prominent GLP-1 RA agent and the first medication receiving market approval. The most commonly described adverse events were GI symptoms for most GLP-1 RA categories, while for tirzepatide social media posts, the most prevalent ones were headache and joint pain. The popularity of these medications and mentions of their adverse events surged in alignment with novel indications, approvals (particularly for children), and celebrity endorsement. We also uncovered 3 distinct clusters of referenced co-occurring adverse events, characterized by (1) somatic or metabolic symptoms (purple), (2) neurological or inflammatory discomfort (pink), and (3) gastrointestinal distress (brown). Together, these findings offer a glimpse into the public’s social media discussions of adverse events related to GLP-1 RA medications. Posts discussing GLP-1 RAs as a class as well as specific posts discussing Mounjaro were most likely to mention adverse events (1138/4885, 23.3% and 868/4031, 21.5%, respectively), while posts discussing the other products (ie, semaglutide, tirzepatide, and Wegovy) included adverse events <10% of the time. However, it is important to note that Mounjaro and Zepbound (both tirzepatide products) were approved during the study period in May 2022 and November 2023, respectively, while Ozempic and Wegovy (both semaglutide products) were approved in 2017 and 2021, respectively. Thus, temporal differences in market access may have impacted the public’s discussion of adverse events for these medications. More recently approved medications may garner more discussion about adverse events, while discussions of medications that have been available and used longer may have shifted toward other topics, such as health insurance coverage or novel indications [25,26]. GLP-1 RA adverse events may be dose dependent and diminish with time. Nevertheless, in our study, we did not collect dose information or include it in our analysis as doses were not consistently posted or available, and it was not possible to assess what dosages of the medications the adverse events were experienced on and attributed to.

GI adverse events, including nausea and vomiting, were the most frequently mentioned for all the drugs, consistent with both clinical trial and social media analysis data [27,28]. Additionally, our study found that Ozempic and all semaglutide medications were more frequently associated with reports of depression and anxiety than other weight loss medications. While our analyses cannot establish a causal relationship or provide a mechanistic pathway, the clustering of depressive symptoms among individuals discussing semaglutide is concerning and builds upon prior literature, raising concerns about an increased risk of suicidal ideation with semaglutide therapy [29,30]. Therefore, it may be important to monitor individuals treated with semaglutide—and other GLP-1 RAs—for mood concerns until more conclusive clinical data are available. Nevertheless, it remains to be determined whether semaglutide impacts mood through its actions on the central nervous system and reward pathways, or whether individuals at high risk for depression are more likely to take semaglutide and share their concerns on social media.

Not surprisingly, we found significant increases in social media posts—both overall and those discussing medication adverse events—corresponding to the broader context during our study period between January 2022 and June 2024. After the FDA approved Wegovy for weight management in children aged 12‐17 years in December 2022 [19], there was a corresponding increase in adverse event mentions in the following months. This trend aligns with a previous study that highlights potential adverse event risks, including GI issues, mental health concerns, and metabolic changes in younger populations [31]. During August 2023, there was an increase in media coverage of GLP-1 RA—particularly of compounded GLP-1 RA products—across social media platforms, concurrent with an FDA warning against use of unapproved GLP-1 RA drugs. This time period also corresponded to a second spike in adverse event mentions as illustrated in Figure 2 [32]. Our findings are consistent with an earlier report that revealed increased patient concerns and discussions about GLP-1 RA adverse events following increased media attention [33]. The next spike in social media discussions was observed after Oprah Winfrey shared that she had used weight loss medication as part of her health and fitness routine in December 2023, which contributed to a 400% increase in semaglutide prescriptions [34]. This surge coincided with a rise in public discourse about associated GI adverse events as well as more severe complications such as pancreatitis and kidney damage [35,36]. Finally, there was an increase in mentions of adverse event concerns after the March 2024 FDA expansion of Wegovy’s approved indication to include the prevention of cardiovascular events in patients with obesity and cardiovascular disease, thereby paving the way for Medicare coverage of Wegovy for patients with obesity but not type 2 diabetes. A subsequent public poll by the Kaiser Family Foundation similarly noted an increase in public discussions of GLP-1 RA adverse events, including GI issues and fatigue [37]. Unlike previous studies, our study uniquely analyzed real-time social media discussions on GLP-1 RA medications, capturing shifts in adverse events mentioned in response to key regulatory and media events.

Previous clinical studies have also examined the adverse event profiles of semaglutide and tirzepatide GLP-1 RAs receptor agonists. For instance, in SURPASS-2, a 40-week phase 3 clinical trial comparing tirzepatide and semaglutide in 1879 participants with type 2 diabetes [10], the most commonly reported adverse events were GI: nausea, diarrhea, vomiting, and reduced appetite. Social media results not only corroborated the high prevalence of these GI adverse events but also exposed some more severe and rare adverse events, such as pancreatitis, which appeared more prominently in social media discussions than in clinical trials. For example, pancreatitis, which was experienced by 0.6% of patients in SUSTAIN-2, was discussed in 2.85% (139/4885) of GLP-RA’s social media posts. Joint pain was an additional adverse event that appeared on social media but is not yet attributed to these medications in clinical trials [38]. Similarly, headache was the most frequently mentioned adverse events mentioned in 1.86% (78/4202) of tirzepatide social media posts. In contrast, a clinical study revealed reduced headache risk in patients with hypertension treated with tirzepatide [39]. These findings warrant further clinical investigation to examine the complex adverse events of tirzepatide on mitigating and exacerbating headaches and other neurological symptoms. Additionally, our study identified 3 unique clusters of adverse event profiles attributed to GLP-1 RA agents. We found co-occurrences between GI adverse events, such as nausea, vomiting, pancreatitis, and GI distress. Anxiety and depression were also highly correlated with each other. These findings build upon an earlier mixed methods study that analyzed Reddit, YouTube, and TikTok to assess the impact of GLP-1 RAs on mental health [7], finding 3 overarching mental health–related themes: insomnia (n=620 matches), anxiety (n=353), and depression (n=204); these mental health conditions were also detected in our analysis. Another study analyzed Twitter, Reddit, PubMed, SIDER, and manufacturer reports to identify clusters of adverse events frequently co-mentioned in the same posts [23]. The most prevalent cluster of co-mentioned adverse events was GI adverse events, corroborating our analytical findings. Next, mental health adverse events were a frequent cluster, aligning with the neurological adverse events cluster in our integrated node network analysis. This study not only leveraged a novel platform, Facebook, to reveal additional insights into relationships between comentioned adverse events but also corroborated the results of previous studies using similar methodologies. Our broader co-occurrence trends reveal broad adverse event categories that can be further explored to analyze individual differences between patients. Further clinical research is necessary to understand the complex relationships of co-occurring adverse events.

### Strengths and Limitations

This quantitative social media analysis study has several strengths. Primarily, it evaluated adverse event profiles attributed by individuals to different GLP-1 RA agents over a 2-year period and across 63,023 posts. Social media posts represent a wide range of individuals, including those from diverse backgrounds who are underrepresented in clinical trials (racial and ethnic minority patients, women of childbearing age, rural residents, etc), in different drug markets, and from all regions of the United States [40]. Facebook is a popular social media platform with 68% of US adults using this digital platform in 2023 [41]. On social media, people have the opportunity to share their real-world experiences, beliefs, concerns, and outcomes and may be more likely to report some experiences they may not report in an in-person study.

However, the study’s limitations must also be taken into consideration. While in general, social media users represent a diverse sample, we do not know the demographic characteristics of the social media users in our specific dataset. Symptoms mentioned in these posts are self-reported, and we cannot discern whether they were personally experienced, represent concerns about potentially experiencing those adverse events, or were posted in response to hearing about adverse events from others. Additionally, social media users may differ from the general US population treated with GLP-1 RA medications. However, more than 7 in 10 Americans use social media to engage with others, receive news, and share information, and the social media user base is increasingly becoming more representative of the broader US population [42]. As such, social media posts about GLP-1 RA medications may represent a combination of the experiences of the broader population taking these medications, those interested in taking these medications, or expressions of concern about these adverse events. They do not solely represent the adverse events reported by the subset of patients with type 2 diabetes or obesity who are prescribed these medications in clinical practice. Furthermore, for those taking medications, we do not know the dosage or the length of time that they were taking these medications. Thus, while social media posts cannot replace clinical trials or large cohort studies, they provide a meaningful, complementary data source to detect adverse events that may warrant further study. Furthermore, this study includes a subset of weight loss medications as identified from Facebook posts. Future studies can expand on this work to examine other drugs used for weight loss and investigate these questions using additional social media platforms. While the analyzed social media posts were in the English language and based in the United States, social media posts in other languages and from other countries can also be analyzed in the future to expand the scope and generalizability of the study’s findings. To construct a list of potential adverse events, we used past literature, clinical expertise, and the SIDER database version 4.1 and did not use the subscription-based product, Medical Dictionary for Regulatory Activities [43]. It would be valuable for future studies to investigate the extent to which findings differ depending on approaches used to identify adverse events. While compounded versions of GLP-1 RAs are not FDA-approved and have been associated with significant adverse drug reactions, this study did not distinguish adverse events from FDA-approved GLP-1 RAs compared with compounded versions of GLP-1 RAs [31]. It would be valuable for future studies to examine differences in reported adverse events of FDA-approved and compounded versions of these drugs.

### Conclusions

Social media provides a space for users to share personal experiences, challenges, and outcomes with weight loss medications in a way that is infeasible to ascertain from other data sources and study designs. Through our comprehensive analysis of Facebook posts during 2022 and 2024, we were able to systematically identify and characterize adverse event profiles attributed to GLP-1 RAs. Using advanced data filtering and extraction techniques, we were able to identify patterns and trends within these data to effectively highlight the unique role of social media in examining the public narrative about increasingly popular weight loss medications. As the rates of GLP-1 RA use continue to grow, it is imperative to investigate their short-term and long-term adverse events and user perceptions of adverse events associated with each drug, along with the complex relationships between concurrent adverse events. Overall, this study enhances our understanding of the nuanced perspectives of the therapeutic landscape, helping facilitate further discovery in this area.

## Supplementary material

10.2196/73619 Multimedia Appendix 1 Supplementary tables and figures displaying additional details regarding glucagon-like peptide-1 receptor agonists and adverse events.

## Abbreviations:

FDA: Food and Drug Administration (United States)
GPP-1 RA: glucagon-like peptide-1 receptor agonist

## References

[1] Singh G, Krauthamer M, Bjalme-Evans M (2022). Wegovy (semaglutide): a new weight loss drug for chronic weight management. J Investig Med.

[2] Whitley HP, Trujillo JM, Neumiller JJ (2023). Special report: potential strategies for addressing GLP-1 and dual GLP-1/GIP receptor agonist shortages. Clin Diabetes.

[3] (2024). National diabetes statistics report. Centers for Disease Control and Prevention (Diabetes).

[4] Neumiller JJ, Bajaj M, Bannuru RR (2025). Compounded GLP-1 and dual GIP/GLP-1 receptor agonists: a statement from the American Diabetes Association. Diabetes Care.

[5] Panic G, Yao X, Gregory P, Austin Z (2020). How do community pharmacies in Ontario manage drug shortage problems? Results of an exploratory qualitative study. Can Pharm J (Ott).

[6] Arillotta D, Floresta G, Guirguis A (2023). GLP-1 receptor agonists and related mental health issues; insights from a range of social media platforms using a mixed-methods approach. Brain Sci.

[7] Zheng Z, Zong Y, Ma Y (2024). Glucagon-like peptide-1 receptor: mechanisms and advances in therapy. Signal Transduct Target Ther.

[8] Sargeant JA, Henson J, King JA, Yates T, Khunti K, Davies MJ (2019). A review of the effects of glucagon-like peptide-1 receptor agonists and sodium-glucose cotransporter 2 inhibitors on lean body mass in humans. Endocrinol Metab (Seoul).

[9] Frías JP, Davies MJ, Rosenstock J (2021). Tirzepatide versus semaglutide once weekly in patients with type 2 diabetes. N Engl J Med.

[10] Chen W, Cai P, Zou W, Fu Z (2024). Psychiatric adverse events associated with GLP-1 receptor agonists: a real-world pharmacovigilance study based on the FDA Adverse Event Reporting System database. Front Endocrinol.

[11] (2024). FDA approves new medication for chronic weight management. FDA.

[12] Gao Y, Duan W, Rui H (2022). Does social media accelerate product recalls? Evidence from the pharmaceutical industry. Inf Syst Res.

[13] Leonard E (2023). Oprah Winfrey reveals she uses weight-loss medication as a “maintenance tool”: “I’m absolutely done with the shaming” (exclusive).

[14] Wreschnig LA, Congressional Research Service (2024). Medicare coverage of GLP-1 Drugs.

[15] Shiffman N, Silverman B CrowdTangle opens public application for academics. Meta Research.

[16] (2024). Transparency Center. CrowdTangle.

[17] (2019). What is a serious adverse event?. US Food and Drug Administration.

[18] Kuhn M, Letunic I, Jensen LJ, Bork P (2016). The SIDER database of drugs and side effects. Nucleic Acids Res.

[19] (2022). News details. Novo Nordisk.

[20] Ring S, Court E, Kresge N (2023). Ozempic’s surging popularity has Novo Nordisk (NVO) struggling to meet demand. Bloomberg.com.

[21] Noguchi Y (2024). Medicare plans can now cover Wegovy for patients at risk of heart disease. NPR.

[22] Aynaud T (2025). taynaud/python-louvain.

[23] Bartal A, Jagodnik KM, Pliskin N, Seidmann A (2024). Utilizing AI and social media analytics to discover adverse side effects of GLP-1 receptor agonists. arXiv.

[24] Shen L, Tai Z, Shen E, Wang J (2023). Graph exploration with embedding-guided layouts. arXiv.

[25] Sidik SM (2024). Ozempic and Mounjaro aren’t the same. here’s how weight-loss drugs compare. Scientific American.

[26] Logan P (2024). On the Increase in Use of GLP-1s blogs.

[27] Yu J, Lee J, Lee SH, Cho JH, Kim HS (2022). A study on weight loss cause as per the side effect of liraglutide. Cardiovasc Ther.

[28] Yao H, Zhang A, Li D (2024). Comparative effectiveness of GLP-1 receptor agonists on glycaemic control, body weight, and lipid profile for type 2 diabetes: systematic review and network meta-analysis. BMJ.

[29] Heaney C (2024). Study links semaglutide and suicidal ideation risk. newsGP.

[30] Schoretsanitis G, Weiler S, Barbui C, Raschi E, Gastaldon C (2024). Disproportionality analysis from World Health Organization data on semaglutide, liraglutide, and suicidality. JAMA Netw Open.

[31] Nowogrodzki J (2024). Should young kids take the new anti-obesity drugs? What the research says. Nature New Biol.

[32] (2024). FDA’s concerns with unapproved GLP-1 drugs used for weight loss. FDA.

[33] Ovalle D, McGinley L (2023). Patients grapple with side effects of popular weight-loss drugs. The Washington Post.

[34] Hopper L (2024). Ozempic popularity among privately insured may boost disparities. USC Today.

[35] Thompson D (2024). Cost keeps many who need them from getting new weight-loss meds.

[36] Hopper L (2024). USC Study: exploding popularity of Ozempic, Wegovy among privately insured patients may worsen disparities. USC Alfred E Mann School of Pharmacy and Pharmaceutical Sciences.

[37] Montero A, Sparks G, Presiado M, Hamel L (2024). KFF health tracking poll may 2024: the public’s use and views of GLP-1 drugs. KFF.

[38] Bliddal H, Bays H, Czernichow S (2024). Once-weekly semaglutide in persons with obesity and knee osteoarthritis. N Engl J Med.

[39] Azzam AY, Essibayi MA, Farkas N (2024). Efficacy of tirzepatide dual GIP/GLP-1 receptor agonist in patients with idiopathic intracranial hypertension. a real-world propensity score-matched study. medRxiv.

[40] Stewart J, Krows ML, Schaafsma TT (2022). Comparison of racial, ethnic, and geographic location diversity of participants enrolled in clinic-based vs 2 remote COVID-19 clinical trials. JAMA Netw Open.

[41] Gottfried J (2024). Americans’ social media use. Pew Research Center.

[42] Atske S (2021). Social media use in 2021. Pew Research Center: Internet, Science & Tech.

[43] (2019). MedDRA. Meddra.org.

