# Supplementary material for: Harnessing Facebook to Investigate Real-World Mentions of Adverse Events of Glucagon-Like Peptide-1 Receptor Agonist (GLP-1 RA) Medications: Observational Study of Facebook Posts From 2022 to 2024
Source: JMIR Infodemiology. 2025 Jul 24;5:e73619. doi: 10.2196/73619 (PMC12289294; doi:10.2196/73619)
Supplement: Multimedia Appendix 1 [file infodemiology-v5-e73619-s001.docx]

Table S1. Prevalent types of GLP-1 RAs receptor agonists.

| Generic name | Brand name | FDA approved for | FDA-indicated adverse event | Date FDA approved | | Sources |
| --- | --- | --- | --- | --- | --- | --- |
| Semaglutide | Ozempic | Type 2 Diabetes | Nausea, vomiting, diarrhea, abdominal pain, constipation | | Dec. 5, 2017 | <https://www.accessdata.fda.gov/drugsatfda_docs/label/2017/209637lbl.pdf> |
|  | Wegovy | Weight Loss | Abdominal distention, eructation, flatulence, gastroenteritis, gastroesophageal reflux disease, nasopharyngitis, fatigue, hypoglycemia, dizziness, headache, dyspepsia, nausea, vomiting, diarrhea, abdominal pain, constipation | | Jun. 4, 2021 | <https://www.accessdata.fda.gov/drugsatfda_docs/label/2022/215256s005lbl.pdf> |
| Tirzepatide | Mounjaro | Type 2 Diabetes | Reduced appetite, dyspepsia, nausea, vomiting, diarrhea, abdominal pain, constipation | | May 13, 2022 | <https://www.accessdata.fda.gov/drugsatfda_docs/label/2022/215866s000lbl.pdf> |
|  | Zepbound | Weight Loss | Nausea, diarrhea, vomiting, constipation, abdominal pain, dyspepsia, injection site reactions, fatigue, hypersensitivity reactions, eructation, hair loss, gastroesophageal reflux disease | | Nov. 8, 2023 | https://www.accessdata.fda.gov/drugsatfda_docs/label/2023/217806s000lbl.pdf |

Table S2. Percentage of medications mentioned within posts about specific adverse events.

| Adverse events | Ozempic | Mounjaro | Tirzepatide | Wegovy | Semaglutide | GLP-1 | Zepbound | | |
| --- | --- | --- | --- | --- | --- | --- | --- | --- | --- |
| Total Posts | 18733 | 17146 | 8527 | 4885 | 4202 | 4031 | 1769 | | |
| Total number of  adverse event  mentions | 2579 | 1486 | 1495 | 1138 | 436 | 868 | 169 | | |
| Nausea | 12.3 | 9.9 | 11 | 11.7 | 13.3 | 13.9 | 13.6 | | |
| Diarrhea | 6 | 4.1 | 4.4 | 5.6 | 4 | 5.6 | 7.1 | | |
| Constipation | 5.5 | 4.4 | 6 | 5.5 | 4.3 | 5.2 | 9.5 | | |
| Vomiting | 11.9 | 17.6 | 3.9 | 15.3 | 6.7 | 9.9 | 6.5 | | |
| Abdominal Pain | 4.3 | 2.2 | 1.4 | 2.3 | 1.5 | 4.5 | 1.2 | | |
| Kidney damage | 0.2 | 0.2 | 0.2 | 0.1 | 0.3 | 0.4 | 0 | | |
| Thyroid tumor | 2.1 | 1 | 0.7 | 1.3 | 1 | 0.7 | 0.6 | | |
| Fatigue | 2.5 | 2.4 | 7.6 | 3.1 | 7.5 | 2.5 | 10.7 | | |
| Dizziness | 0.7 | 0.2 | 0 | 1.1 | 1 | 0.7 | 0.6 | | |
| Gastrointestinal | 18.9 | 27.8 | 4.4 | 19.8 | 6.4 | 18.5 | 9.5 | | |
| Loss of Appetite | 0.2 | 0 | 0 | 0.3 | 0.1 | 0.4 | 0 | | |
| Indigestion | 0.2 | 0.6 | 0 | 0 | 0.3 | 0.2 | 0 | | |
| Dehydration | 0.3 | 0.2 | 0 | 0.2 | 0.8 | 0.4 | 0 | | |
| Headache | 0.9 | 0.8 | 17.9 | 0.9 | 7.5 | 1.8 | 1.8 | | |
| Swelling | 0.4 | 0.2 | 0.9 | 0.5 | 1.6 | 0.1 | 0 | | |
| Hypertension | 0.9 | 1.3 | 4.8 | 1.7 | 3.6 | 2.8 | 7.7 | | |
| Allergies | 0.3 | 0.2 | 0.2 | 0.2 | 0.9 | 0.1 | 0 | | |
| COPD | 0.3 | 0.1 | 0 | 0 | 0 | 0.4 | 0 | | |
| Hypoglycemia | 3.1 | 1 | 1.6 | 2.2 | 2.1 | 2.5 | 1.2 | | |
| Anxiety | 2.7 | 2.2 | 3 | 2.4 | 4 | 3.4 | 5.3 | | |
| Depression | 6.4 | 2.2 | 3.9 | 9 | 10.2 | 3.7 | 6.5 | | |
| Heart Palpitations | 0.1 | 0.2 | 0.2 | 0 | 0.2 | 0 | 0 | | |
| Insomnia | 0.2 | 0.6 | 0.5 | 0.1 | 0.5 | 0.4 | 0 | | |
| Muscle Cramps | 0 | 0 | 0 | 0 | 0.2 | 0 | 0 | | |
| Hair Loss | 5.9 | 9.2 | 3.4 | 5.2 | 3.4 | 4.2 | 10.1 | | |
| Pancreatitis | 6.4 | 5.1 | 2.8 | 6.1 | 3.5 | 12.2 | 4.1 | | |
| Vision Changes | 0.2 | 0.3 | 0.5 | 0.2 | 0.2 | 0.4 | 1.2 | | |
| Gallbladder Issues | 2.4 | 0.3 | 0.2 | 2.4 | 0.3 | 1.1 | 0.6 | | |
| Dry Mouth | 0 | 0 | 0.2 | 0 | 0.1 | 0 | 0 | | |
| Sweating | 0.5 | 0.2 | 0.2 | 0.3 | 0.6 | 0.3 | 0 | | |
| Tingling Sensation | 0 | 0 | 0 | 0 | 0.1 | 0 | 0 | | |
| Increased Heart Rate | 0.1 | 0.1 | 0.2 | 0.2 | 0.1 | 0 | 0 | | |
| Irregular Menstrual Cycles | 0.5 | 0.2 | 0.2 | 0.3 | 0.6 | 0.3 | 0 | | |
| Jaundice | 0 | 0 | 0 | 0 | 0.1 | 0 | 0 | | |
| GERD | 0.2 | 0.1 | 0 | 0.1 | 0.1 | 0.4 | 0 | | |
| Muscle Weakness | 0.1 | 0.2 | 0.2 | 0.1 | 0.1 | 0.2 | 0 | | |
| Joint Pain | 0.2 | 0.5 | 16.3 | 0.1 | 6.5 | 0.9 | 0.6 | | |
| Elevated Blood Sugar Levels | 0 | 0 | 0 | 0 | 0 | 0 | 0 | | |
| Seizures | 0.2 | 0 | 0 | 0 | 0.3 | 0 | 0 | | |
| Liver Damage | 0 | 0 | 0 | 0 | 0.1 | 0 | 0 | | |
| Rash | 1 | 0.7 | 0.9 | 0.3 | 3.4 | 0.7 | 0.6 | | |
| Sore Throat | 0.1 | 0 | 0 | 0.2 | 0 | 0.2 | 0 | | |
| Restlessness | 0 | 0 | 0 | 0 | 0 | 0.1 | 0 | | |
| Erectile Dysfunction | 0.1 | 0 | 1.1 | 0.1 | 0.7 | 0.1 | 0 | | |
| Persistent Cough | 0.9 | 2.5 | 0 | 0 | 0 | 0 | 0 | | |
| Hives | 0.3 | 0.1 | 0.2 | 0.1 | 0.1 | 0.5 | | 0 | |
| Back Pain | 0 | 0.2 | 0 | 0 | 0.5 | 0 | | | 0 |
| Shortness of Breath | 0 | 0 | 0 | 0.3 | 0.1 | 0 | | | 0 |
| Blood Clots | 0.1 | 0.1 | 0.2 | 0.1 | 0.1 | 0.4 | | | 0 |
| Fever | 0.3 | 0.3 | 0.2 | 0.3 | 0.3 | 0.2 | | | 1.2 |
| Mood Swings | 0.6 | 0.2 | 0.7 | 0.9 | 1.6 | 0.3 | | | 0 |

Percentage = (Number of Posts About a Specific Medication Mentioning the Adverse Event / Total Number of Posts Mentioning the Adverse Event) × 100

Figure S1. Frequency bar graph of adverse event mentions ≥ 75 for weight loss medications.

**
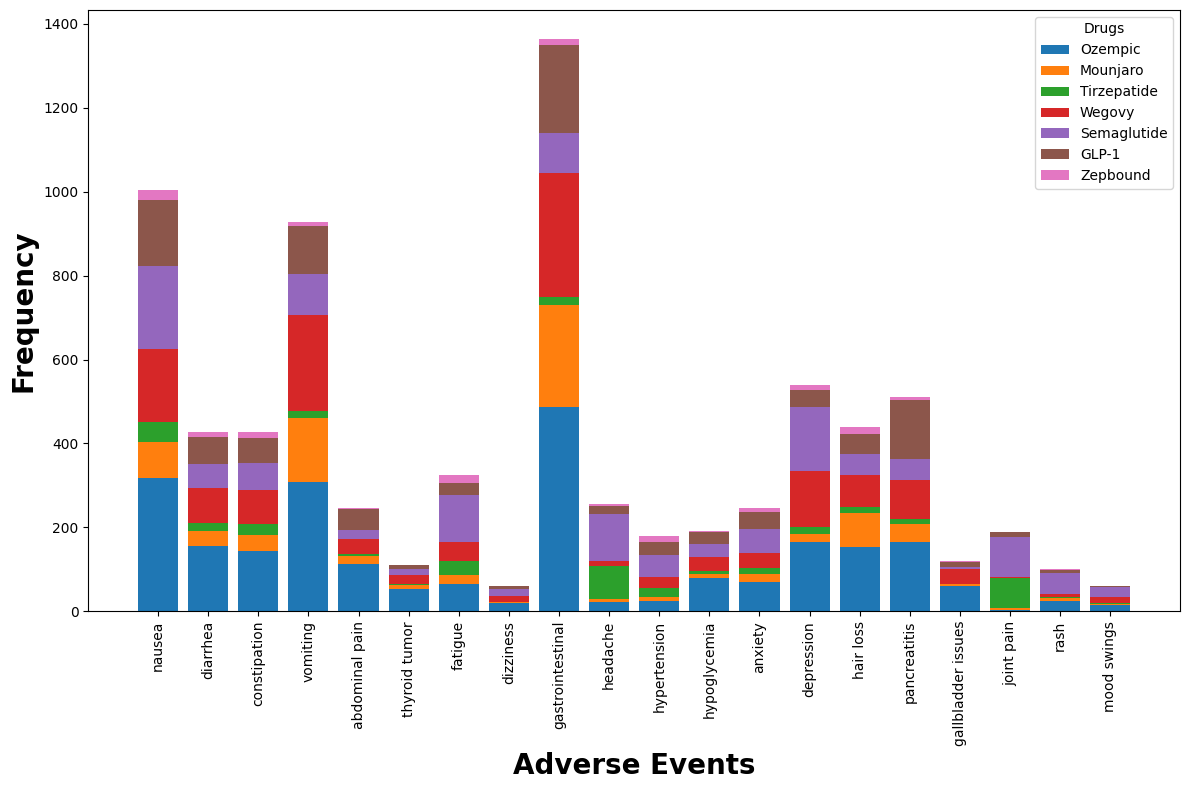
**

Figure S2. Temporal time series analysis of the percentage of adverse event mentions with total drug mentions.

**
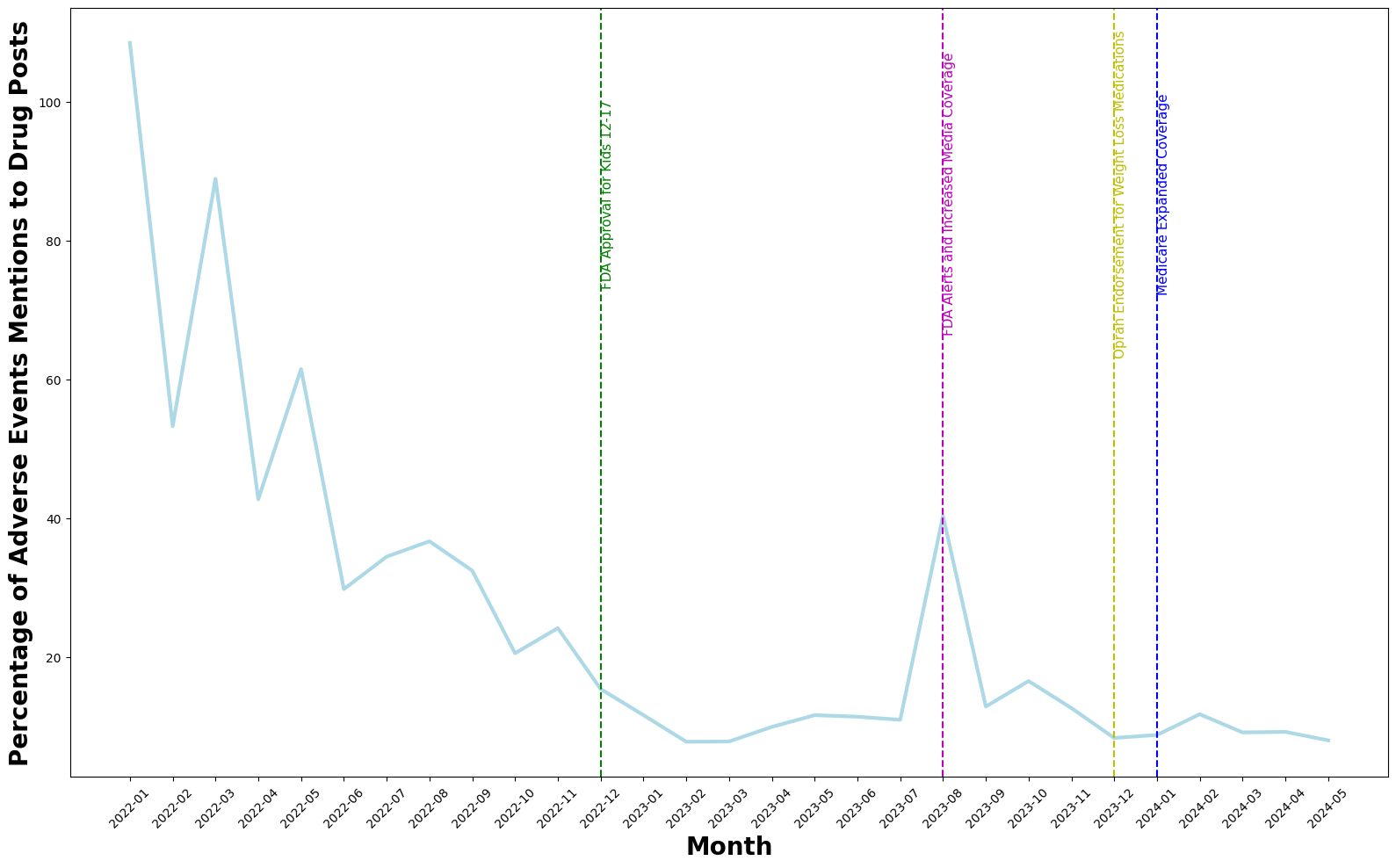
**

Table S3. Counts and percentages of adverse events to total drug mentions.

| **Month** | **Total adverse event mentions** | **Total drug mentions** | **Adverse event percentage** |
| --- | --- | --- | --- |
| 2022-01 | 67 | 59 | 113.56 |
| 2022-02 | 36 | 62 | 58.06 |
| 2022-03 | 59 | 63 | 93.65 |
| 2022-04 | 38 | 89 | 42.70 |
| 2022-05 | 105 | 109 | 96.33 |
| 2022-06 | 81 | 158 | 51.27 |
| 2022-07 | 84 | 122 | 68.85 |
| 2022-08 | 73 | 161 | 45.34 |
| 2022-09 | 61 | 182 | 33.52 |
| 2022-10 | 69 | 317 | 21.77 |
| 2022-11 | 92 | 373 | 24.66 |
| 2022-12 | 82 | 482 | 17.01 |
| 2023-01 | 131 | 1061 | 12.35 |
| 2023-02 | 75 | 916 | 8.19 |
| 2023-03 | 115 | 1374 | 8.37 |
| 2023-04 | 154 | 1324 | 11.63 |
| 2023-05 | 186 | 1459 | 12.75 |
| 2023-06 | 223 | 1822 | 12.24 |
| 2023-07 | 175 | 1429 | 12.25 |
| 2023-08 | 772 | 1841 | 41.93 |
| 2023-09 | 173 | 1193 | 14.50 |
| 2023-10 | 205 | 1103 | 18.59 |
| 2023-11 | 177 | 1219 | 14.52 |
| 2023-12 | 133 | 1321 | 10.07 |
| 2024-01 | 318 | 2106 | 15.10 |
| 2024-02 | 223 | 1687 | 13.22 |
| 2024-03 | 241 | 2381 | 10.12 |
| 2024-04 | 239 | 2346 | 10.19 |
| 2024-05 | 254 | 2681 | 9.47 |
